# Improving the Functionality of Intra-Operative Nerve Monitoring During Thyroid Surgery: Is Lidocaine an Option?

**DOI:** 10.14740/jocmr2025w

**Published:** 2015-02-09

**Authors:** Ramasamy Govindarajan, Ajay Shah, Vemuru Sunil Reddy, Vellore Parithivel, Saiganesh Ravikumar, Dave Livingstone

**Affiliations:** a Department of Anesthesia (A division of North American Partners in Anesthesia), Bronx Lebanon Hospital Center, 1650 Grand Concourse, Bronx, NY 10457, USA; b Department of Surgery, Bronx Lebanon Hospital Center, 1650 Grand Concourse, Bronx, NY 10457, USA; c Emory University, Atlanta, GA, USA

**Keywords:** Intra-operative nerve monitoring, Thyroid surgery, Parathyroid surgery, Recurrent laryngeal nerve paralysis, IV lidocaine infusion

## Abstract

Intra-operative nerve monitoring (IONM) is rapidly becoming a standard of care in many institutions across the country. In the absence of neuromuscular blocking agents to facilitate the IONM, the depth of anesthesia required to abolish the laryngo tracheal reflexes often results in profound hemodynamic instability during surgery, necessitating the use of large doses of sympathomimetic amines. The excessive alpha and beta adrenergic effects exhibited by these agents are undesirable in the presence of cardiovascular co-morbidities. Trying to strike a balance frequently results in an unsatisfactory intra-operative course. In the course of the near total thyroidectomy performed on a 60-year-old female, we employed lidocaine infusion at 1.5 mg/kg/hour following a bolus dose of 1 mg/kg. The troublesome laryngo tracheal reflexes were successfully blunted and we were able to moderate the depth of anesthesia resulting in stable hemodynamics. A bispectral index monitor was employed to guard against “recall” and a train of four monitor was used to ensure the absence of inadvertent neuromuscular blockade. During the surgery, there was loss of signal on the left recurrent laryngeal nerve (RLN). The signal strength was restored by rotating the endotracheal tube on its long axis to realign the electrode with the vocal cords under Glidescope^®^ visualization.

## Introduction

The role of intra-operative nerve monitoring (IONM) in thyroid surgery remains a controversial subject for surgeons regardless of surgical training and background [1]. It is not designed to visualize the recurrent laryngeal nerve (RLN), but to allow intra-operative assessment of RLN function as well as to establish a prognosis in patients developing RLN paralysis [2-4]. Because of the simplicity of design and cost effectiveness, recording of electromyography (EMG) of vocal cords using surface electrodes integrated or attached to the endotracheal tube (ETT) is perhaps the most widely used technique of IONM. Complete functionality of the IONM depends on near total laryngeal relaxation and reflex suppression without the use of neuromuscular blocking agents (NMBAs). The depth of anesthesia required to meet that criteria often leads to hemodynamic instability. We used intravenous (IV) infusion of lidocaine which allowed us to provide good operating and monitoring conditions without significant hemodynamic fluctuations.

## Case Report

The patient was a 60-year-old female with past medical history of valvular heart disease, hypertension, dyslipidemia and glaucoma. Her past surgical history included cesarean section, tubal ligation, hysterectomy and mitral valve repair. She presented with nontoxic multinodular goiter and was planned for near total thyroidectomy. Other than night time palpitations, she denied having chest pain and reported exercise tolerance of five blocks with no symptoms. She quit smoking 6 years ago after 30 years of “a few cigarettes a day”. She admitted to the use of alcohol socially but denied the use of recreational drugs. Her “at home” medications included losartan potassium 50 mg PO daily, metoprolol succinate ER 25 mg PO daily, aspirin 81 mg (delayed release) PO once a day and timolol 0.5% eye drops once a day. Her heart rate was 62/min, with regular rate and rhythm and her blood pressure was 118/82 mm Hg. Rest of her physical examination was within normal limits. Her EKG tracings were normal sinus rhythm and preoperative echocardiogram documented an ejection fraction of 51% with no wall motion abnormalities. Her airway was assessed to be grade 2 Mallampati oropharyngeal classification. She took her daily dose of metoprolol at 6 am on the morning of the surgery.

Dragonfly Single Channel Laryngeal Surface Electrode (Electrode LSE 500Ms; Neurovision Medical Products, Ventura, CA, USA) was applied to a #7 cuffed ETT (Medline Industries, Mundelein, IL, USA) according to the manufacturer’s instructions. The EMG sensor setup was tested as per their guidelines as well. In addition to standard monitors, a right radial arterial line was placed under local analgesia for closer hemodynamic monitoring. The patient was pre-oxygenated and then induced with a bolus of midazolam 2 mg and fentanyl 1.25 μg/kg followed by etomidate 0.25 mg/kg and succinylcholine 1.2 mg/kg intravenously. Direct laryngoscopy and endotracheal intubation proceeded as planned. Esmolol in titrated boluses was used to blunt the sympathoadrenal responses during the intubation. Anesthesia was maintained with 50:50 mixture of air in oxygen and 1 - 2 minimum alveolar concentration of sevoflurane. IV infusion of propofol 50 μg/kg/min was titrated to effect for maintenance. The placement of the electrode plates touching the vocal cords was verified under vision with a Glidescope^®^, after positioning the patient with the desired neck extension.

Within a few minutes of commencement, the surgery was frequently held up by episodes of coughing and “bucking” on the ETT. Increasing the depth of anesthesia to counteract these troublesome reflexes resulted in intense bradycardia and profound hypotension, necessitating the use of large doses of sympathomimetic amines and anti-cholinergics. These medications resulted in a myriad of side effects: phenylephrine, worsening the bradycardia; ephedrine, while unable to improve the blood pressure, resulted in sinus arrhythmia with occasional premature ventricular complexes and glycopyrrolate barely denting the heat rate. Continued use of them, it was feared, will result in adverse hemodynamic consequences with minimal benefits, if any.

It was decided to start an IV infusion of lidocaine at the rate of 1.5 mg/kg/h after a bolus dose of 1 mg/kg. The surgery was allowed to recommence 15 min after the start of the infusion. The laryngo-tracheal reflexes holding up the surgery gradually dissipated and the surgery progressed as planned. We were able to titrate down the IV and inhalational anesthetics as well as the sympathomimetic support without encountering adverse hemodynamic fluctuations. The functionality of the IONM was checked as necessary and visually reconfirmed by the identification of the RLN throughout the procedure. A BIS monitor (Model 1 A 2000; Aspect Medical Systems, Newton, MA, USA) was connected to guard against “recall” during lighter planes of anesthesia and a train of four (TOF) monitor (Microstim Plus, Neurotechnology, Houston, Texas, USA) was employed to guard against inadvertent neuromuscular blockade (NMB). During the course of the surgery, there was loss of signal (LOS) on the left RLN after initial identification and later visual confirmation. The ETT position was checked with the aid of the Glidescope^®^, and the ETT was rotated in its long axis to align the electrode plate with the left vocal cord. The signal strength was restored and further surgery proceeded uneventfully. Lidocaine infusion was tapered down and discontinued upon extubation. During the surgery, 75 μg of fentanyl was used in divided doses to sustain basal narcosis.

The patient stayed overnight in the post anesthesia care unit (PACU) for observation. Her vital signs remained stable. She received two doses of morphine IV (4 mg each) and 4 mg of ondansetron IV during her 14-h stay in PACU before being discharged home.

## Discussion

Several studies have documented that routine identification of the RLN with IONM has decreased the rates of permanent RLN palsy [5-7]. But its role in reducing the frequency of RLN injury remains controversial [8-10]. While the overall incidence of nerve palsy is low, when it occurs it is a devastating lifelong handicap [11, 12]. When bilateral RLN dissection is planned, RLN monitoring is particularly useful to limit the risk of bilateral RLN paralysis. Two-stage thyroidectomy following functional recovery of damaged RLN can then be proposed [2]. After studying 15,403 nerves at risk, Thomusch et al concluded that, when the IONM signal is unchanged after the resection, the surgeon can be reassured that postoperative vocal cord function is normal in 99.6% of cases [7]. Where the IONM signal is absent or abnormal, 30-45% of the patients will develop vocal cord dysfunction postoperatively [7].

The successful deployment and data analysis from the IONM depends upon the complete laryngeal relaxation and reflex suppression. The IONM causes strong contractions of the vocal cords and is an independent cause of laryngeal alterations [13]. The surgical procedure is a strong factor of stress to the larynx with a potentially traumatic manipulation at the level of the larynx [13]. Complete functionality of the IONM constitutes before the full acceleromyographic recovery of the NMB at the adductor pollicis muscle [14], as the laryngeal muscles recover faster than the peripheral muscles [15]. The anesthetic technique chosen should ensure absolute laryngeal reflex suppression as the effect of the short acting depolarizing muscle relaxant is wearing off immediately following intubation, until preferably the extubation. Be it total intravenous anesthesia or inhalation-based anesthesia, the depth required for reflex suppression can cause profound cardiovascular instability. While the attempts to titrate down the anesthetic depth can cause resurfacing of the troublesome laryngeal reflexes, attempts to counter the side effects with sympathomimetic agents can themselves result in a myriad of undesirable hemodynamic consequences. The resulting hemodynamic fluctuations can result in adverse outcomes in patients with significant cardiovascular co-morbidities.

In our case we chose to suppress the laryngo tracheal reflexes by employing IV infusion of lidocaine. Injected intravenously, lidocaine is variably effective in blunting the hemodynamic response to tracheal intubation [16]. IV liodcaine decreases the intracellular Ca^2+^ concentration in airway smooth muscle, decreases myofilament Ca^2+^ sensitivity and has been shown to suppress coughing and prevent reflex broncho constriction [16]. Laryngoscopy with or without intubation, provokes a sympathoadrenal response. These events are especially detrimental in individuals who have limited myocardial reserve because of age or disease; these include geriatric population and those with significant coronary arteriosclerosis as in diabetes mellitus, sustained hypertension, angina, ischemic heart disease and cardiac dysrhythmias [17]. Administration of lidocaine IV 1.5 mg/kg attenuates the hyperdynamic response to laryngoscopy and intubation [17].

IV lidocaine has analgesic, anti-hyperalgesic and anti-inflammatory properties. Local anesthetics can reduce the postoperative inflammatory response in several ways such as blocking neural transmission at the site of tissue injury and thus attenuating neurogenic inflammation. In addition because of anti-inflammatory properties of their own, they may inhibit the migration of granulocytes and release of lysosomal enzymes, consequently leading to a decreased release of pro-inflammatory cytokines. Pro-inflammatory cytokines can induce peripheral and central sensitization, leading to pain augmentation (hyperalgesia) [18]. The remarkably comfortable post operative course that our patient experienced in the PACU can be attributed to the salutary effect of lidocaine on pain and inflammation.

Because of its plasma half life of 8 min, an IV bolus of 1 - 1.5 mg/kg lidocaine must be followed by infusion of 1 - 4 mg/min in adults in order to maintain therapeutic levels of 1.5 - 4 μg/mL [19, 20]. Lidocaine side effects have never been reported in its intended use, probably because the first clinical signs of toxicity occur at considerably high blood concentrations (> 5 μg/mL), which did not occur even when IV lidocaine was given continuously over 14 days [21]. Lidocaine infusion at the rate of 1.5 mg/kg/h following a bolus of 1.5 mg/kg has been employed in a newborn to perform an intra-operative wake-up test during neurosurgery [22].

LOS during IONM after initial identification and visual confirmation is quite often due to migration of the electrode encased ETT either “too far in” or “too far out” during inadvertent extension or flexion of neck during surgical manipulations, resulting in loss of contact (LOC) with the vocal cords. The traction exerted on the ETT by the connecting tubing and the breathing circuit can cause the ETT which is secured in the middle, to rotate in its long axis either to the right or left. The “C” shaped electrode with the opening of the “C” facing anteriorly can lose contact with either of the vocal cords depending on the direction of the axial rotation, as happened in our case. Image scope examination of the ETT and repositioning as necessary will restore the lost signal in the course of the surgery.

### Conclusion

Although the overall incidence of nerve palsy is low, it results in a devastating life-long handicap when it occurs [11, 12]. While IONM is not an alternative to visual nerve identification (VNI), IONM may lend itself as a routine adjunct to the gold standard of VNI [11]. Rising intolerance for physician error, potential legal issues and medical insurers can increase intra-operative stress [11]. IONM can to a large extent mitigate this stress. Improving the functionality of the IONM can increase its sensitivity and further enhance the value of this useful monitoring tool. By employing IV lidocaine infusion, we were able to keep the troublesome laryngo tracheal reflexes at bay, thereby providing good operating and monitoring conditions as well as stable hemodynamic parameters under lighter plane of anesthesia. By not having to administer significant doses of sympathomimetic amines to support the cardiovascular system, we have avoided their undesirable side effects. Finally the beneficial influence of lidocaine on pain and inflammation, we believe, contributed to the stable postoperative course and greatly enhanced patient satisfaction during her stay in the PACU.

## Author Note

The study was presented in part at the Southeastern Surgical Congress Annual Scientific Meeting, in Savannah, GA, USA, in February 2014.
